# Visual outcome and complications in Ab-externo scleral fixation IOL in aphakia in pediatric age group

**DOI:** 10.12669/pjms.294.3791

**Published:** 2013

**Authors:** Isra Ahmed Bhutto, Ghulam Qadir Kazi, P.S Mahar, Umair Ahmed Qidwai

**Affiliations:** 1 Dr. Isra Ahmed Bhutto, FCPS, Assistant Professor, Isra Postgraduate Institute of Ophthalmology, Al-Ibrahim Eye Hospital, Karachi, Pakistan.; 2 Prof. Ghulam Qadir Kazi, FRCS, Professor, Isra Postgraduate Institute of Ophthalmology, Al-Ibrahim Eye Hospital, Karachi, Pakistan.; 3 Prof. P.S Mahar, FRCS, FRC Ophth, Professor, Isra Postgraduate Institute of Ophthalmology, Al-Ibrahim Eye Hospital, Karachi, Pakistan.; 4 Dr. Umair Ahmed Qidwai, FCPS, Senior Registrar, Isra Postgraduate Institute of Ophthalmology, Al-Ibrahim Eye Hospital, Karachi, Pakistan.

**Keywords:** Astigmatism, Complication, Scleral fixation

## Abstract

***Objective:*** To assess the visual outcome and complications in patients after Ab-externo scleral fixation of intraocular lens in pediatric age group (15 years or less).

***Methods:*** This quasi experimental study was conducted at Isra Postgraduate Institute of Ophthalmology, Al-Ibrahim Eye Hospital, Karachi, from January 2012 to December 2012. All cases included were worked up according to the protocol. All patients underwent Ab-externo scleral fixation of IOL under general anesthesia. Patients were followed up at 1^st^day, 1^st^week, 1^st^month, 2^nd^month and 3^rd^month. Complete eye examination including best-corrected visual acuity and complications were noted on each visit.

***Results:*** Thirty patients were included in the study, with mean age of 8.6 years (±3.93569). Most of the patients, 20 (66.7%), had visual acuities of 6/18 or better. No complication was seen in 18 (60%) of the patients intra operatively while soft eye was observed in 7 (23.3%) of the patients. Another complication noted was vitreous hemorrhage, which was seen in 5 (16.7%) patients. Most common post-operative complication was Uveitis followed by astigmatism. Lens dislocation and iris abnormalities were seen in only one patient. Most of the patients showed significant visual improvement after surgery.

***Conclusion:*** Ab-externo scleral fixation of an IOL was found to be safe and showed favorable postoperative results with fewer complications.

## INTRODUCTION

Intraocular lens (IOL) implantation to correct the aphakia offers superior visual rehabilitation in comparison to aphakic spectacles or contact lens.

In the absence of capsular support; anterior chamber lenses, iris fixated lenses and scleral fixated intraocular lenses may be considered.^1^^,^^2^ The placement of IOL in the posterior chamber rather than anterior or iris fixated lenses reduces the risk of various complications, like keratopathy, damage to anterior chamber angle structure, pupillary block glaucoma, hyphema, uveitis, iris chafing, dislocationandpseudophakodonesis.^3^

Additionally, positioning lens closer to the rotational center of the eye, just anterior to the vitreous face, may reduce the centrifugal forces on the lens and stabilize the ocular contents, thereby decreasing the probability of complications such as iritis, cystoids macular edema (CME) and retinal detachment.^4^^,^^5^ Another advantage of positioning the lens closer to the nodal point and center of the eyes is the superior optical properties of the lens in this position.^6^

There are two surgical techniques, namely Ab-interno (inside out) and Ab-externo (outside in). The Ab-interno technique involves the passage of needle from the inside of the eye to the outside through the sclera.^7^^,^^8^ The Ab-externo technique involves the passage of a needle from the outside of the eye to the inside through the sclera. In the Ab-externo method; scleral fixation of IOL exactly in the ciliary sulcus can be achieved.^9^ With this method, the surgeon’s view is never obscured. All the manipulation occurs in the iris plane. The surgeon can thus decrease the risk of vitreous hemorrhage.^10^ Retinal detachment and lens mal position by avoiding the potential inaccuracies of suture placement those are inherent to the Ab-interno technique.^11^^,^^12^

## METHODS

 The study included 30 eyes of 30 consecutive patients who underwent Ab-externo scleral fixation of IOL during January 2012 to December 2012 at Isra Postgraduate Institute of Ophthalmology, Al-Ibrahim Eye Hospital, Karachi. All patients were selected from the pediatric ophthalmology clinic of Al-Ibrahim Eye Hospital. The patients were in pediatric age group (15 years or less). Those patients with visually significant ocular pathologies involving angle structure, cornea, retina, macular and optic nerve were excluded. A written informed consent was taken prior to procedure. The detailed history of each patient was taken about any major illness, in general, and other ophthalmic problems in particular. A detailed ophthalmic examination was carried out including charting of best corrected visual acuity using Snellen’s notation, bio microscopic examination of anterior segment, measurement of IOP and fund us examination. All children have biometry preformed before the procedure.

***Surgical Procedure: ***All procedures were performed under general anesthesia. After preparing the patient a conjunctival peritomy was created at 3 O’clock and at 9 O’clock position. A partial-thickness limbal-based scleral flap measuring 3mm long and 2mm wide was fashioned at the same position. A 7mm corneal incision was made superiorly at 12 O’ position and anterior chamber (AC) was entered through 3.2mm knife. After forming the AC with viscoelastic substance, a complete anterior vitrectomy was performed.

After reformation of AC, a straight needle carrying a 9-0 polypropylene suture was placed through the 9O’clock sclera bed parallel to the iris and 0.8mm to 2mm posterior to the posterior surgical limbus. The needle tip was passed through the sulcus and behind the iris until it was visualized behind the pupil. In a similar manner, a 28-gauge needle was inserted through the 3o’clock sclera bed. The barrel of the 28-gauge needle was inserted into the eye and the syringe was withdrawn from the eye (the syringe carried with it the straight needle and suture). A loop of this suture was withdrawn through the corneal sclera wound. The loop of suture was cut, and securely tied one end to the superior haptic and the other to the inferior haptic.

The lens was inserted into the sulcus, and rotated into position while removing slack from the attached sutures. Second 9-0 polypropylene sutures were used on a half-circle needle to take a short bite in the 3O’clock scleral bed just anterior to the first suture’s exit. The long end of the second polypropylene suture was tied to the hybrid suture; in a square knot with fourth rows. The same steps were followed in the 9O’clock scleral bed. Scleral flaps were closed, and the conjunctiva re-approximated.

All patients were followed after one week and monthly for three months after the surgical procedure during each visit, best corrected visual acuity (BCVA) was checked along with anterior segment examination using slit lamp. These findings were noted on proforma and analyzed subsequently.

***Statistical analysis: ***All calculation were done by SPSS Version 17.0 frequencies percentages were calculated for qualitative variables like, gender, complications, visual outcome for pre and postoperative. Me-Nemar test was used to compare the difference between pre-operative best corrected visual outcome (BCVA) and postoperative (BCVA) with level of significance 0.05.

## RESULTS

Thirty patients were included in the study, with mean age of 8.6 years (±3.93569). Minimum age of the patient’s included was 2 years while the maximum age was 15 years. Out of 30 patients, 19 (63.3%) were male and 11 (36.7%) were female. Most of the patients, 12 (40%), were in the age group of 5 to 10 years while minimum no of patients, 8 (26.7%), were in the age group of less than 5 years. Most of the patients, 20 (66.7%), had visual acuities of 6/18 or better. Comparison of visual acuities before and after surgery is shown in Table-I. No complication was seen in 18 (60%) of the patients intra operatively while soft eye was observed in 7 (23.3%) of the patients. Another complication noted was vitreous hemorrhage, which was seen in 5 (16.7%) patients (Table-II). Most common post-operative complication was Uveitis followed by astigmatism. The IOL dislocation and iris abnormalities were seen only in one patient respectively. The frequency and percentage of postoperative complication are shown in Table-III.

**Table–I:** Visual acuities before and after surgeries

	*Before surgery*		*After surgery*	
	*Frequency*	*%*	*Frequency*	*%*
6/6-6/18	9	30.0	20	66.7
6/24-6/36	15	50.0	8	26.7
6/60 or less	6	20.0	2	6.7
Total	30	100.0	30	100.0

**Table-II:** Intra operative Complications

	*Frequency*	*Percent*
Soft eye	7	23.3
Vitreous hemorrhage	5	16.7
No complication	18	60.0
Total	30	100.0

**Table-III:** Postoperative complications

	*Frequency*	*Percent*
Astigmatism	9	30.0
Lens dislocation	1	3.3
Corneal edema	2	6.7
Uveitis	10	33.3
CME	2	6.7
Iris abnormalities	1	3.3
Exposed sutures	5	16.7
Total	30	100.0

## DISCUSSION

Optical rehabilitation of a patient with monocular aphakic presents a therapeutic challenge. Aphakic glasses, because of magnification and anisoconia cannot be prescribed. Almost 75% of patients in this group are unable to tolerate contact lenses. The viable options therefore include, epikeratophakia, anterior chamber IOL implant, iris fixated intraocular lens and sclera fixated posterior chamber IOL implants. Among them scleral fixated PC IOL implant can provide minimum magnification of the image as compared to other options.^13^

In our study, the post-operative best corrected visual acuity of 6/12 or better was achieved in 30 (66.7%) patients who underwent Ab-externo scleral fixation of IOL. This is comparable with Lee and Yuen’s work^14^ who reported best corrected visual acuity of 6/12 or better in 19 (76%) out of 25 cases. Ghanem and colleagues^15^^,^^16^ reported postoperative BCVA of 6/9 or better in 10(71.43%) out of 14 eyes undergoing scleral fixation of IOL. Similarly, Ozdekandco-workers^17^ reported the improvement of postoperative BCVA 6/12 or better in 14 eyes (93.3%) undergoing scleral fixation of IOL.

The most common post-operative complication in our series was Uveitis and astigmatism in 10 and 9 eyes respectively. The minimum astigmatism was-2.00DC and maximum was - 3.50DC. (Mean astigmatism was - 2.42DC) in this study. The cause of astigmatism was large corneal incision or tight sutures and IOL decent ration. Ghanem and colleagues^15^ also reported astigmatism as most frequently occurring complication in 3 eyes (21.4%). Similarly, Sasahara and Kiryu^18^ reported astigmatism in 12 eyes (13%).

Due to iris manipulation while doing scleral fixation of IOL, we noticed anterior uveitis in 10 eyes which is comparable with the results of Kwong et al and Kanigowska K.^19^^-^^20^

Due to the unavailability of foldable scleral fixating IOL and endoscope, we did not use them in this study. The outcome could be improved further by taking care of certain measures like using foldable IOL instead of rigid PMMA (polymethyl methacrylate) IOL as the former can be inserted by giving small incision as compared to later one. This will ultimately reduce the postoperative astigmatism.^21^ Similarly to insert the IOL without tilting and preventing its decent ration, the haptics should be placed precisely into the ciliary sulcus that can be accomplished by using an endoscope.^22^

The positive findings of this study are that our results are comparable to other studies done in different regions, proving the efficiency of the procedure. The lack of serious complication makes it an effective alternate to other methods of optical correction with inadequate capsular support.

***Limitations of the study: ***There are certain limitations of this study. This study does not have an epidemiological value as incidence and prevalence of aphakic within adequate capsular support cannot be ascertained. This is because the sampling technique was non probability convenience.

## CONCLUSION

Our results suggest that Ab-externo scleral fixation of an IOL was found to be a safe procedure showing a favorable postoperative visual outcome in aphakic eyes in pediatric age group.
